# Emerging trends of online assessment systems in the emergency remote teaching period

**DOI:** 10.1186/s40561-022-00199-6

**Published:** 2022-03-29

**Authors:** Arif Cem Topuz, Eda Saka, Ömer Faruk Fatsa, Engin Kurşun

**Affiliations:** 1 Department of Computer Engineering, Engineering Faculty, Ardahan University, 75000 Ardahan, Turkey; 2 Department of Computer Technologies, Vocational School of Technical Sciences, Bayburt University, 69000 Bayburt, Turkey; 3 Ministry of Education, 03000 Afyonkarahisar, Turkey; 4 Department of Computer Education and Instructional Technologies, Kazım Karabekir Education Faculty, Atatürk University, 25000 Erzurum, Turkey

**Keywords:** Online assessment system, Online exam, Online measurement and evaluation, Online proctoring system, Trends of online assessment systems

## Abstract

The COVID-19 pandemic caused many educational institutions in the world to switch to the distance education process, and this process was called "Emergency Remote Teaching". This urgent transition process has caused many problems in educational environments. One of the problems is the subject of measurement and evaluation. Along with the pandemic, many institutions have used various online assessment systems to make measurements and evaluations online, and researchers have conducted research on these online assessment systems. This research focus on the features of the online assessment systems and aims to examine the trends towards the prominent features of the online assessment systems in the Emergency Remote Teaching period. For this purpose, the prominent online assessment systems have been determined by systematically analyzing academic studies published in 2020, and answers have been sought to the following research questions: (1) which platforms they support, (2) which security features they have, and (3) what common features they have. Identifying trends in the characteristics of online assessment systems is expected to guide practitioners, decision-makers, researchers, and system developers in the process of selecting and/or developing an online assessment system for use in online measurement and evaluation.

## Introduction

It is an undoubted fact that exams are extremely important to achieve teaching goals (Brown et al., 2013; Fuchs & Fuchs, 1984). Online education applications have increased in recent years, both due to the COVID-19 pandemic and the development of Information and Communication Technologies (ICT). Online exams also tend to increase in parallel with this situation since most of the exams, quizzes, tests, and many other measurement and evaluation tools have been moved to online platforms in Emergency Remote Teaching (ERT) period.

The concept of ERT emerged as a result of the crisis experienced during the COVID-19 pandemic. It includes workarounds rather than a permanent plan. Unlike planned distance education activities, it aims to produce fast, practical, reliable, and flexible solutions to problems (Hodges et al., 2020). The need for secure online assessment systems has been felt more especially during the COVID-19 period. Therefore, online assessment tools should also have features that can provide solutions to the problems in the ERT period.

Security issue has been a common problem for online exams (Barthel, 2016; Butler-Henderson & Crawford, 2020; Dadashzadeh, 2021). This damages the reputation of training programs and reduces the value of the diploma in the eyes of potential employers (Carrell et al., 2008; Dadashzadeh, 2021). As a solution, security-enhanced online assessment systems are used to prevent cheating and fraud (Foster & Layman, 2013; Slusky, 2020; Tomasi et al., 2011).

Secure online assessment systems are in constant development in parallel with the progress in ICT, such as integration of artificial intelligence, analyzing data with image processing, utilizing data mining technique, widespread of the internet, and development of network infrastructure. These systems employ various methods to prevent and/or detect cheating. For instance, Ryu et al. (2020) have expressed that single-factor authentication was replaced by a multimodal biometric system in online assessment systems and proposed a multibiometric system that uses face recognition and keystroke dynamics for continuous authentication. Arnò et al. (2021) have conducted two case studies by analyzing the features of existing commercial online assessment systems and proposed developing an automatic surveillance system to be used in online assessments that performs continuous face recognition and also allows manual surveillance. Li et al. (2021) have offered a technique that simultaneously sent questions to students and worked on optimization to perform an online exam, and they obtained positive results. Considering many such features of online assessment systems, selecting/developing an online assessment system has become difficult for those who would like to select/develop an online assessment system.

With the increasing need for online assessment tools in the ERT period, the need for guidelines for the selection of these tools has increased (Rahim, 2020). Therefore, this research has been carried out in order to see the current state of online assessment tools as of 2020 and to guide the selection/development processes of these tools. Conducting research on determining trend in the features of online assessment systems can help/guide (i) institutions and decision-makers in their choice of online assessment system, (ii) researchers in the academic studies by showing the gaps about online assessment systems, and iii) system developers in defining the features that an online assessment system should have. In addition, it is thought that identifying trends in online assessment systems can contribute to predict the future of online assessment systems.

First of all, studies examining the features of online assessment systems in the literature have been examined in detail in order to understand the trends of current online assessment systems. The next section explains the reviewed studies in the literature and the contribution of this research to the literature.

## Related work

It has been observed that studies examining the features of online assessment systems, which tend to become widespread rapidly after COVID-19 period, are quite limited. One of the basic studies on examining the features of online assessment systems belongs to Foster and Layman (2013). Their study compares the features of eight secure online assessment systems if terms of proctoring features, lockdown features, authentication options, webcam features, and overall security capability. They have lighted the future of secure online assessment systems by suggesting the following methods: usage of webcams, the interaction between the examinees and the test administration, analyzing the testing events instantly, and evaluating the level of security risk by taking advantage of existing data sources (such as demographics, test stakes, and testing history).

Another study on features of online assessment systems has been conducted by Karim and Shukur (2016). They have focused on online authentication methods for online exams and have proposed a new model (called Preferences Based Authentication) after examining the existing authentication methods both used by five commercial online assessment systems and mentioned in the literature. They have classified the existing authentication techniques under the following four categories: knowledge-based authentication, possession-based authentication, biometric-based authentication, and other authentication mechanisms (location, timestamp, IP address, etc.). Butler-Henderson and Crawford (2020) have addressed authentication and security, interface design, and technology issues in online assessment systems as parts of their review search. They have gathered various issues regarding online assessment systems mentioned by existing literature.

Slusky (2020) has conducted research by reviewing the 20 online proctoring systems and summarized important features of the systems. Features of the systems have been classified under the following categories: access control, compliance, control (AI-assisted, blockchain), controls (keystrokes, blocked ports, etc.), detection, interaction, platform, proctoring, recognition, recording, and test content. Another study, conducted by Hussein et al. (2020), has identified popular online proctoring systems by reviewing the studies on online exam proctoring and performing desk-based research of popular online proctoring tools. Then, they have evaluated the selected 4 popular systems for further understanding the functionalities of the systems. They have compared the features of online assessment systems considering the following characteristics: proctoring features, lockdown features, authentication options, and webcam features.

Arnò et al. (2021) have examined the features of 29 prominent commercial online assessment systems and compared automatic and live proctoring online assessment systems. They have used Google and Google Scholar to identify the online assessment systems with the following keywords: proctoring exams, online exams, and proctoring system. Then, they have identified and compared the features of the systems in terms of the following categories: LMS integration, scalability, Android/iOS secondary device support, authentication functions, lockdown functions, monitoring functions, force completion of the exam, live chat support, GDPR compliance, free, user-friendly, platforms, plugin/browser extension, needs client installation, internet connection, open source, and proctoring category.

The existing literature shows that various researchers have identified prominent online assessment systems by reviewing academic studies and have compared the examined systems considering the characteristics of them. At this point, the purpose of this research differs from the existing literature by focusing on features of the prominent online assessment systems instead of comparing the systems. Therefore, this research is important as it will highlight the features of the systems instead of suggesting a system and will guide those who will select/develop an online assessment system that suits their needs. This research aims to reveal the emerging trends of the prominent online assessment systems as of 2020 and to guide the selection/development processes of online assessment systems by synthesizing the characteristics towards (1) supported platforms, (2) security features used, and (3) common features of the systems. For this purpose, answers were sought for the following research questions:

1. Which platforms do the online assessment systems support?
2. Which security features stand out in the online assessment systems?
3. What are the common features of online assessment systems?

## Research method

This research determines the current status of online assessment systems, the need for which was felt more in the ERC period, by examining their features. To determine the prominent online assessment systems, this research systematically analyzed the articles published in 2020 on online assessment systems. The list of online assessment systems has been obtained by following the procedures proposed by Kitchenham (2004). These procedures include three stages, namely: 1) planning, 2) conducting, and 3) reporting. Moreover, results were reported considering an additional guideline proposed by Moher et al. (2010), namely PRISMA (Preferred Reporting Items for Systematic Reviews and Meta-Analyses). Thus, this section reports the data collection and analysis process within the guidelines mentioned above and shows the list of reviewed online assessment systems.

## Data collection and analysis

The features of the prominent online assessment systems that were used or mentioned by the published articles are the data of this research. The data sources to identify these prominent systems are the articles published in 2020, and the data sources to determine the characteristics of the systems are the documents/texts published on the websites of these systems. To collect and analyze the data, the following five stages proposed by Kitchenham (2004) were performed: (1) identification of research, (2) selection of primary studies, (3) study quality assessment, (4) data extraction and monitoring, and (5) data synthesis.

**Fig. 1:**
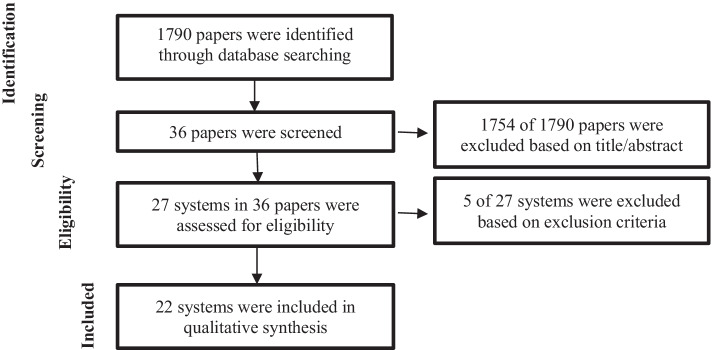
PRISMA flow diagram

Firstly, to identify the prominent online assessment systems in 2020, a search strategy was determined, including the selection of a search tool and the identification of search keywords. The authors chose Google Scholar since (a) it is the most comprehensive search tool for finding academic studies (Martín-Martín et al., 2021) and (b) it avoids selection bias by making the data source widely available. Then, singular and plural (*) combinations of the following keywords were identified to reach relevant studies via advanced search feature of Google Scholar: online, exam*, assessment*, system*, proctoring. Once searching process was completed, the authors found 1790 studies with the identified keywords. Secondly, to select relevant articles among 1790 studies, the following inclusion criteria were identified: written in English, published in a peer-reviewed journal, full-text with open-access, published in 2020, and referring to an online assessment system. By reading the titles and abstracts of 1790 studies (Moher et al., 2010), 1754 studies were excluded since they did not meet the inclusion criteria. Therefore, the remaining 36 articles were included as they met the inclusion criteria. Then, to select online assessment systems mentioned in these 36 studies, system inclusion criteria were identified as follows: name of online assessment system (a) used or (b) mentioned in the study. Thirdly, while evaluating the quality of 36 studies, 2 authors read the studies in order to avoid detection bias. Both authors read the full texts of the 36 studies to identify the names of the online assessment systems considering the system inclusion criteria. Thus, 27 systems were identified and included in this research as the same 27 online assessment systems were identified by both authors. At this point, Arnò et al. (2021) reached 29 online assessment systems as a result of their search using similar keywords without any date limitation. The fact that the number of systems obtained as a result of the search results of two different studies is close to each other supports the reliability of the data. Fourthly, to extract irrelevant systems among the 27 systems, exclusion criteria were identified as follows: (a) not fully developed systems, (b) only one module related to online exams, and (c) already included in the dataset because it has multiple names. Thus, 5 of 27 systems were excluded considering those exclusion criteria. Fifthly, this research synthesized the features of the 22 online assessment systems through a data collection form adapted from Foster and Layman (2013). The data collection form was filled in by visiting the websites of the systems in January 2021. All these procedures were visualized by the PRISMA flow diagram in Fig. 1.

## Reviewed online assessment systems

In this research, 22 online assessment systems have been examined to answer the research questions. Table 1 shows the names of all these online assessment systems with their website addresses.

**Table 1:** Reviewed online assessment systems

No	Names	Website addresses	No	Names	Website addresses
1	Eklavvya	eklavvya.in	12	Proctorfree	proctorfree.com
2	Eproctoring	eproctoring.com	13	Proctorio	proctorio.com
3	Examity	examity.com	14	Proctortrack	proctortrack.com
4	Examsoft	examsoft.com	15	ProctorU	proctoru.com
5	Examus	examus.net	16	PSI-Live Proctoring	psionline.com
6	Honorlock	honorlock.com	17	Respondus	respondus.com
7	Iris invigilation	irisinvigilation.com	18	Safe Exam Browser	safeexambrowser.org
8	Kryteriononline	kryteriononline.com	19	Smowl	smowl.net
9	Loyalist	loyalistexamservices.com	20	Speed Exam	speedexam.net
10	Mettl	mettl.com	21	Talview	talview.com
11	ProctorExam	proctorexam.com	22	Tegrity	tegrity.com

Table 1 shows the list of analyzed online assessment systems and websites. Analysis results are discussed in the next section.

## Results and discussion

This research aims to reveal the trends in the features of online assessment systems. Results were synthesized within the framework of three research questions and discussed under the following themes: RQ1) supported platforms, RQ2) security features, and RQ3) common features. Frequency (f) values shown in the figures and tables show how many of the 22 examined systems have the relevant characteristics.

### RQ1: Platforms supported by the prominent online assessment systems

**Fig. 2:**
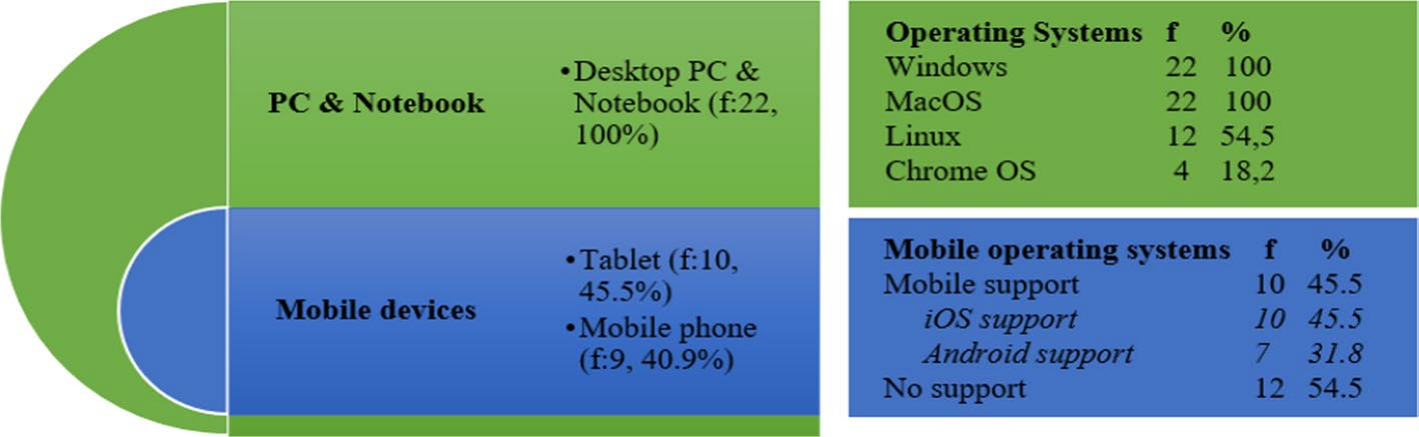
Device, operating system, and mobile operating system support

Features of the platforms supported by online assessment systems are synthesized under five categories considering the study conducted by Foster and Layman (2013), namely: (1a) devices, (1b) operating systems, (1c) mobile operating systems, (1d) web browsers, and (1e) e-learning platforms. Figure 2 shows the supported platforms for devices, operating systems, and mobile operating systems. The following subtitles discuss the results regarding each category.

#### Supported devices

Figure 2 illustrates that all the online assessment systems support both Desktop PCs (f = 22, 100%) and Notebooks (f = 22, 100%). However, only half of those can support mobile devices, namely Tablets (f = 10, 45.5%) and Mobile phones (f = 9, 40.9%). The distribution of tablet devices by mobile operating systems is as follows: iOS-based tablets (f = 10, 45.5%) and Android-based tablets (f = 7, 31.8%). The distribution of Mobile phones by mobile operating systems is as follows: iOS-based phones (f = 9, 40.9%) and Android-based phones (f = 7, 31.8%). Thus, this result reveals that all of the prominent online assessment systems can be run on Desktop PCs and Laptops, but almost half of these current systems can be run on mobile devices.

Even though students tend to use mobile devices in online assessments (Karadeniz, 2011), it has been understood that some of the existing systems are insufficient to enable students to attend exams via mobile devices. The fact that online assessment systems do not support mobile devices may be due to (i) the difficulties in preventing cheating on mobile devices and (ii) the frequent usage of mobile devices as a cheating tool by students (Jones et al., 2013; Kanchan, 2021; King et al., 2009). In addition, students have stated that they do not want to use devices with low resolution and small screen size in online assessment systems (Heng et al., 2005; Joy et al., 2005). Despite the various studies (Kamble & Ghorpade, 2021; Tiong & Lee, 2021) that focused on preventing cheating in online assessment systems, there is a need to develop online assessment systems where students can participate in online exams both from mobile devices and in a supervised manner. Therefore, future studies can focus on developing online assessment systems that are both compatible with mobile devices and secure against cheating.

#### Supported operating systems

Figure 2 reveals the distribution of operating systems supported by the leading online assessment systems in 2020, as follows: Windows (f = 22, 100%), MacOS (f = 22, 100%), Linux (f = 12, 54.5%), and Chrome OS (f = 4, 18.2%). This result shows that prominent online assessment systems can work with 100% compatibility with both Windows and MacOS. This distribution is overlap with the Desktop Operating System Market Share Worldwide report (StatCounter Global Stats, 2021), and that report explains that online assessment systems are compatible with the operating systems at a total rate of ~ 95% (Windows:75%, MacOS:16%, Linux:2.2%, and Chrome OS:2%). The reason why online assessment systems are developed as being compatible with Windows and MacOS may be due to the facts that: (i) the number of operating system users may have been taken into account when prioritizing development, and (ii) some of the online assessment systems operate as application-based software rather than as web browser-based software, and not all application-based software is compatible with all operating systems yet, such as Linux and Chrome OS. Tüfekci et al. (2013) stated that online assessment systems should be run on common operating systems and web browsers. Therefore, this research suggests that future online assessment systems can be developed to work on/via web browsers in order to be compatible with much more operating systems.

#### Supported mobile operating systems

Analyzing the mobile operating system support of the leading online assessment systems reveals that a little more than half of the online assessment systems (f = 12, 54.5%) do not have mobile operating system support yet (Fig. 2). Fortunately, 10 of 22 online assessment systems (45.5%) support mobile operating systems, including the iOS platform (f = 10, 45.5%) and/or Android platform (f = 7, 31.8%). At this point, although Android devices are more affordable for students (Kaiiali et al., 2016) and there are more Android users than iOS users (Garg & Baliyan, 2021), it is interesting that the number of systems with iOS support is higher than that of Android support. If system developers can develop web browser-based online assessment systems instead of app-based ones, it can be the solution to the mobile OS compatibility problem.

#### Supported web browsers

Online assessment systems are not compatible with all web browsers. Therefore, compatible/supported web browsers have been determined in this category and are shown in Table 2.

**Table 2:** Supported web browsers

Web Browsers	f	%
Chrome	17	77.3
Firefox	12	54.5
Internet Explorer	10	45.5
Safari	7	31.8
Their own browser	5	22.7
Opera	4	18.2
Yandex	2	9.1

As shown in Table 2, the distribution of web browsers supported by the analyzed online assessment systems is as follows: Chrome (f = 17, 77.3%), Firefox (f = 12, 54.5%), Internet Explorer (f = 10, 45.5%), Safari (f = 7, 31.8%), Opera (f = 4, 18.2%), and Yandex (f = 2, 9.1%). In addition, 5 of 22 systems (22.7%) use their own browser (f = 5, 22.7%). This result goes along with the existing literature since existing studies (Nelson et al., 2020; Woo et al., 2020) report that Chrome, Firefox, and Internet Explorer are the most widely used web browsers. In addition, Garcia Laborda et al. (2016) have stated that they prefer Chrome because it enables students to navigate comfortably, and students with disabilities do not have problems. Similarly, Topa and Karyda (2018) note in their study that users do not prefer a different browser other than Chrome due to their perception of reliability.

To detect and prevent cheating, online assessment systems usually need to access users' personal data, such as webcam images, screenshots, and GPS coordinates. Therefore, future research may explore which features of web browsers can be used by online assessment systems to detect and prevent cheating and how.

#### Supported e-learning platforms

Integration of e-Learning platforms can facilitate data transfer from/to the online assessment systems. Therefore, this category reveals the platforms supported by the online assessment systems, and Table 3 presents the obtained e-Learning platforms.

**Table 3:** Supported E-learning platforms

E-learning platforms	f	%
Moodle	17	77.3
Blackboard	16	72.7
Canvas	16	72.7
D2L (Brightspace)	12	54.5
Sakai	6	27.3
Open edX	5	22.7
Schoology	2	9.1
Opigno	2	9.1
Other	3	13.6
Undefined	3	13.6

Analyzing the supported e-Learning platforms has revealed that the majority of the online assessment systems support Moodle (f = 17, 77.3%), Blackboard (f = 16, 72.7%), Canvas (f = 16, 72.7%), and D2L (f = 12, 54.5%). Other supported e-Learning platforms are as follows: Sakai (f = 6, 27.3%), Open edX (f = 5, 22.7%), Schoology (f = 2, 9.1%), Opigno (f = 2, 9.1%), other (f = 3, 13.6%), and undefined (f = 3, 13.6%).

This result shows that Moodle, Blackboard, and Canvas are the most frequently used e-learning systems that work well with prominent online assessment systems. According to Yorulmaz and Can (2020), the reason why Moodle is preferred so much is as follows: it is free and open-source, and its plugins are developed and shared by many developers. Besides, Moodle is used by 302,000,000+ users in more than 240 countries (Moodle Statistics, 2021). Even though Learning Management System (LMS) integration is not a mandatory requirement for an online assessment system, it can still facilitate the identification of courses, students, and instructors in the online assessment systems. At this point, Arnò et al. (2021) have stressed the difficulty in the identification of this kind of information in the existing online assessment systems.

### RQ2: Security features of the prominent online assessment systems

The second theme determines the security features of the online assessment systems. The concept of security in online assessment is about the features included in the system to detect/prevent cheating and/or fraud. Security features have been synthesized and discussed under the following five categories: 2.a) Authentication, 2.b) Restrictions, 2.c) Monitoring, 2.d) Data Analysis, and 2.e) Intervention Methods.

#### Authentication methods used in online assessment systems

Authentication methods enable the online assessment system to verify the identity of the user/student to ensure the correct person has taken the exam. This category exhibits authentication methods used in the analyzed online assessment systems, and these authentication methods have been classified according to the time when they were applied, namely: before, during, and after the exam. Table 4 shows all the authentication methods along with the times they were applied in the online assessment systems.

**Table 4:** Authentication methods used in online assessment systems

Authentication methods	Before the exam		During the exam		After the exam	
	f	%	f	%	f	%
Government-issued ID	19	86.4	2	9.1	0	0
Face recognition	16	72.7	3	13.6	1	4.5
Voice recognition	9	40.9	3	13.6	0	0
One-time password	9	40.9	0	0	1	4.5
Keystroke analysis	3	13.6	2	9.1	0	0
Challenge questions	3	13.6	0	0	0	0
Eye-tracking	1	4.5	1	4.5	0	0
Fingerprint recognition	1	4.5	0	0	0	0

Table 4 illustrates that the majority of the online assessment systems authenticate the users/students before the exam begins through government-issued ID (f = 19, 86.4%) and/or face recognition (f = 16, 72.7%). Besides, other authentication methods used before beginning the exam are as follows: voice recognition (f = 9, 40.9%), one-time password (f = 9, 40.9%), keystroke analysis (f = 3, 13.6%), challenge questions (f = 3, 13.6%), eye-tracking (f = 1, 4.5%), and fingerprint recognition (f = 1, 4.5%). Several online assessment systems use five of these authentication methods during the exam, including: government-issued ID (f = 2, 9.1%), face recognition (f = 3, 13.6%), voice recognition (f = 3, 13.6%), keystroke analysis (f = 2, 9.1%), and eye-tracking (f = 1, 4.5%). In addition, two online assessment systems also use authentication methods after the exam to verify the user/student, namely: face recognition (f = 1, 4.5%) and one-time password (f = 1, 4.5%).

When the authentication methods are evaluated from a broad perspective, it can be seen that the systems mostly use the biometric (i.e., face and voice) data of the users/students for authentication. Moini and Madni (2009) stated in their study that traditional authentication methods are not secure and that biometric authentication methods should be used in the process (before the exam, during the exam, and after the exam). Similarly, Adetunji et al. (2018) draw attention to the use of biometric data related to the reliability of the methods used in identification. Considering authentication: The fact that biometric authentication methods are not preferred may be due to the necessity of the hardware, its expensiveness, and the opinion that not everyone can afford this hardware. In addition, it has been seen that the most commonly used authentication methods are the verification methods that web-based online assessment systems can easily perform. Fingerprint reader, iris reader, etc. Biometric authentication methods, which are less used, are generally preferred in online exam centers, which is seen as a new trend. The cost of hardware, the benefit of many, etc., appear to have such benefits. Many online assessment systems offer services to private and public institutions, such as setting up an online exam center or taking exams at their own online exam centers.

One of the least preferred authentication methods in the research is security (f = 3) questions. Similarly, Renaud and Just (2010) have revealed in their study that it is a safer method to question by choosing from among the pictures instead of using the security question method. Agulla et al. (2008) have stated in their study that it is not possible to determine a completely error-free identity. For this reason, it is thought that there is a tendency towards newer technological identification methods. In addition, the use of biometric data is low, students have the right to keep their profile information private and confidential (Marais et al., 2006), people do not want this data to be shared without their permission (May et al., 2012), and so on may be caused for various reasons.

#### Restriction methods used in online assessment systems

Online assessment systems use restriction methods and disable various features on a user's device in order to prevent cheating. Table 5 shows the restriction methods used in the analyzed online assessment systems towards cheating.

**Table 5:** Restriction methods used in online assessment systems

Restriction methods	f	%
Disabling copy/paste	15	68.2
Disabling exit from full-screen	13	59.1
Disabling print-screen	12	54.5
Disabling right click	12	54.5
Disabling additional application running	11	50
Disabling printing	11	50
Disabling control keys of browser	10	45.5
Disabling function keys	10	45.5
Disabling communication applications/tools	10	45.5
Disabling simultaneous multi-entry to test	10	45.5
Disabling virtual machine usage	9	40.9
Disabling remote desktop connection	9	40.9
Disabling exit from test	8	36.4
Disabling combinations of shortcut keys	7	31.8
Disabling desktop and tools	5	22.7
Disabling menu and icons	4	18.2
Disabling multi-screen usage	2	9.1

According to Table 5, the most commonly used restriction methods in assessment systems are disabling copy/paste (f = 15, 68.2%), blocking exit from full-screen (f = 13, 59.1%), turning off print-screen function (f = 12, 54.5%), deactivating right click (f = 12, 54.5%), preventing additional software from being run (f = 11, 50%), and enabling unprinting (f = 11, 50%).Although online assessment systems have many more features such as these restrictions, researchers are still investigating new restriction methods since students still cheat (Ebaid, 2021; Jia & He, 2021). Therefore, more research is needed on how effective the systems are at preventing fraud with those restrictions.

Examining the disabled features in online assessment systems has revealed that some restrictions (such as hiding desktop, menu, and icons) require users to install an additional application. However, the need to set up a program or a web browser plugin may prevent an assessment system from operating platform-independently. Therefore, future studies can consider the restriction methods synthesized in this research to deal with fraud and cheating.

#### Monitoring methods used in online assessment systems

Security-enhanced online assessment systems enable instructors to monitor participants to ensure security in exams. This category has synthesized the monitoring/surveillance methods used in the analyzed online assessment systems under four sub-categories, namely: a) Semi-automatic Monitoring (Machine and Human Proctoring), b) Full-automatic Monitoring (Machine Proctoring), c) Live Monitoring (Human Proctoring), and d) Record Monitoring. These four classifications can be explained as follows: In a semi-automated monitoring system, the assessment system detects fraud and then a human decides whether the students are cheating or not. In a fully automated monitoring system, both detection and decision of cheating processes are performed by the machine (online assessment system). Live monitoring systems enable a human proctor to watch/follow all students’ personal data (such as images, screens, and answers) during the exam. Record monitoring systems enable the human proctor to monitor recorded personal data of students at any time after the exam. Table 5 shows the numbers and percentage distributions of monitoring methods used in the online assessment systems below.

Table 6 shows that the most preferred two monitoring methods in online assessment systems are semi-automatic monitoring (n = 13, 59.1%) and full-automatic monitoring (n = 4, 18.2%), respectively. Other monitoring methods are live monitoring (n = 2, 9.1%) and record monitoring (n = 2, 9.1%), and the monitoring method is not specified in one system (n = 1, 4.5%). The reason why automatic and semi-automatic monitoring systems are more preferred may have stemmed from the affordability of those systems (Jose, 2016). In addition, the reason why semi-automatic monitoring is the most preferred method instead of full-automatic monitoring may be that artificial intelligence and image processing technologies are considered insufficient for fraud detection. Besides, artificial intelligence and human intelligence are still discussed in terms of critical thinking skills (Spector & Ma, 2019). Claiming that a student cheated is a serious accusation, so the calculation error for the claim must be zero. After the fraud detection of the semi-automatic monitoring system, the human proctor makes the final decision, which means two-step verification. However, regardless of which method is used, existing literature shows that the number of unsuccessful students is higher in supervised exams than in unsupervised exams (Daffin & Jones, 2018; Richardson & North, 2013; Stack, 2015). This means that even just conducting an online exam under surveillance can contribute to reducing cheating.

**Table 6:** Monitoring methods used in online assessment systems

Monitoring method	n	%
Semi-automatic monitoring (machine and human proctoring)	13	59.1
Full-automatic monitoring (machine proctoring)	4	18.2
Live monitoring (human proctoring)	2	9.1
Record monitoring	2	9.1
Unspecified	1	4.5

On the other hand, this research has revealed a misunderstanding between semi-automatic monitoring and full-automatic monitoring. Some of the analyzed online assessment systems state on their web pages that the surveillance is done fully automatically, but when the working principles of these systems are examined, it is understood that these systems just collect students' personal data and then present those data to a human (not the system) to decide whether there is cheating or not. To be called full-automatic monitoring/proctoring, it is not enough to receive image data from the user; it should also be determined whether there is a fraudulent situation in that image data.

#### Data analyzed in online assessment systems

To provide security in online exams, online assessment systems can analyze various data during the online exam. This category has classified the data collected for this purpose in the analyzed assessment systems, and Table 7 shows the collected data with frequency and percentage distributions.

**Table 7:** Data analyzed by online assessment systems

Collected data	f	%
Video record	19	86.4
Image record	18	81.8
Voice record	18	81.8
Screen record	18	81.8
IP address	10	45.5
Keyboard usage record	6	27.3
Time record	6	27.3
Geolocation record	1	4.5
Web traffic record	1	4.5

Table 7 shows that online assessment systems frequently collect the following data to provide security in online exams: video (f = 19, 86.4%), image (f = 18, 81.8%), voice (f = 18, 81.8%), screen record (f = 18, 81.8%), and IP address (f = 10, 45.5%). Other data is as follows: keyboard usage record (f = 6, 27.3%), time record (f = 6, 27.3%), geolocation record (f = 1, 4.5%), and web traffic record (f = 1, 4.5%). This result has revealed that most online assessment systems use biometric data to detect and/or prevent cheating. Besides, the majority of the prominent online assessment systems analyze video, image, voice, and/or screen records. The reason why these online assessment systems are included in the selected studies may be that they analyze various data to ensure security in online exams.

In some countries, collecting/analyzing/storing users' biometric data is a sensitive subject that requires high attention by law. Therefore, it would be better for researchers/developers to pay attention to laws when using biometric data for authentication in an online assessment system. For instance, according to the Personal Data Protection Law of Turkey (2016), if users’ personal data will be used for any analysis, then users should be informed about which data will be collected/analyzed/stored and what analysis will be performed. Otherwise, this kind of law on the processing of personal data prevents online assessment systems from using biometric data for authentication in many other countries (Topuz et al., 2021).

#### Remote intervention methods used in online assessment systems

Remote intervention features empower proctors or systems to intervene with users when security breaches are detected during the exam. This category has classified the remote intervention methods used in the analyzed assessment systems, and Table 8 shows the remote intervention methods with frequency and percentage distributions.

**Table 8:** Remote intervention methods used in online assessment systems

Remote intervention methods	f	%
Showing a message/announcement/warning	9	40.9
Finalizing the test	5	22.7
Freezing and resuming the test	4	18.2
Undefined	7	31.
No intervention	4	18.2

Table 8 has revealed that less than half of the prominent online assessment systems (f = 9, 40.9%) have remote intervention features. These limited features consist of showing a message/announcement/warning (f = 9, 40.9%), finalizing the test (f = 5, 22.7%), and freezing and resuming the test (f = 4, 18.2%). In addition, there is no intervention method in 4 of the analyzed systems, and no intervention method is specified in 7 of the analyzed systems. Rather than allowing cheating and then punishing it, intervening in a timely manner may be more effective in reducing cheating. At this point, it would be the correct method to intervene during the exam. However, showing messages very often, using inappropriate words in the messages, and freezing/resuming the exam can distract students during the exam (Singer, 2015). Therefore, the intervention methods should be carefully planned and applied. Future studies can address this issue to determine appropriate intervention methods that can be used without disrupting students during online exams.

### RQ3: Common features of the prominent online assessment systems

The third theme contributes to literature by determining the common features of the prominent online assessment systems. Common features have been synthesized under the following four categories: (3a) question types, (3b) support services, (3c) hardware or software requirements, and (3d) integration methods.

#### Question types used in online assessment systems

This category has been classified into the types of questions supported by the leading online assessment systems. Table 9 shows the frequency and percentage distributions of question types below.

**Table 9:** Distribution of question types used in online assessment systems

Question types	f	%
Multiple choice	14	63.6
Essay (long answer)	11	50
True/false	11	50
Short answer	9	40.9
Option matching	9	40.9
Fill-in-the-blank	8	36.4
Multiple response	7	31.8
Drag&drop	4	18.2
Calculation	3	13.6
Audio/video recording	2	9.1
Code snippet question	2	9.1
Sequence	2	9.1
Select from list	1	4.5
Numeric	1	4.5
Diagram type	1	4.5

Table 9 has revealed that the most commonly used five question types are as follows: multiple choice (f = 14, 63.3%), essay (f = 11, 50%), true/false (f = 11, 50%), short-answer (f = 9, 40.9%), and option matching (f = 9, 40.9%). Başal (2015) stated in his study that students are satisfied with the fact that the choices in multiple-choice questions are prepared and that they have the opportunity to compare them with other questions and choices. Bayazıt (2013) expressed that they used multiple-choice question types in his research due to the creation of clearer and clearer tasks. In addition, the fact that these types of questions are easy to evaluate by the system and the evaluation made is economical (Ridgway et al., 2004) may be another reason.

Richardson et al. (2002) drew attention to the necessity of doing more studies on developing problem-solving skills in computer-based assessment and increasing the use of graphics and animations in these questions. Therefore, in future studies, it can be investigated to what extent the question types determined to be prominent in online assessment systems meet the stated need.

#### Support services in online assessment systems

Online assessment systems include various support services to help students during the online exams. Table 10 shows the support services available in the analyzed online assessment systems.

**Table 10:** Support services in online assessment systems

Support services	f	%
Frequently asked questions	20	90.9
Help documents	17	77.3
Technical support module	16	72.7
Live chat module	14	63.6
Demo module/page	13	59.1
System test page	13	59.1
Calculator	12	54.5
Customization of the interface	2	9.1

When Table 10 is examined, it is understood that the supportive services offered are generally aimed at solving the technical problems that the users will encounter during the exam. The support services most commonly found in online assessment systems are Frequently Asked Questions (f = 20, 90.9%), Help Documents (f = 17, 77.3%), and Technical Support Module (f = 16, 72.7%). According to previous research (Ozden et al., 2004; Wen & Tsai, 2006), students want a help module, an application module, an explanation module, and a calculator in their assessment systems. Furthermore, the fact that students want an explanation section where they can take notes (Bloom et al., 2018; Khan & Khan, 2019; Ozden et al., 2004) and that their grades are saved explains why these supportive tools are preferred in online assessment systems. In addition, students' willingness to use calculators in online assessment systems (Bloom et al., 2018) coincides with the findings of the current research.

The possibility of live chat with the lecturer/supervisor has been seen in semi-supervised online assessment systems and mostly in technical matters, warnings, etc. with the supervisor, has been used at times. The low frequency of live chat with a lecturer/supervisor may be due to its low usability, especially in crowded classrooms. When the literature is examined, it has been found that students want to get feedback from the instructors, but they complain that online exams reduce their interaction with the instructor (Khan & Khan, 2019).

The system test page allows the student to test the device on which they will participate in the exam before the exam. Thus, students will not encounter surprises during the exam. In addition, in the examination conducted within the scope of this study, it has been seen that there are demo pages in some exam systems, but many processes must be performed to use these demo pages. It can be said that more functional demo pages can be useful to eliminate this problem.

#### Hardware or software requirements in online assessment systems

Students need various types of hardware or software to be able to attend online exams via online assessment systems. Table 11 illustrates the requirements that students should have in order to participate in exams on prominent online assessment systems.

**Table 11:** Hardware or software requirements in online assessment systems

Requirements	f	%
Webcam	20	90.9
Microphone	20	90.9
Internet connection	20	90.9
Web browser plug-in installation	8	36.4
Web browser installation	5	22.7
Desktop application installation	3	13.6

Table 11 shows that the features needed in prominent online assessment systems are as follows: webcam (f = 20, 90.9%), microphone (f = 20, 90.9%), internet connection (f = 20, 90.9%), web browser plug-in installation (f = 8, 36.4%), web browser installation (f = 5, 22.7%), and desktop application installation (f = 3, 13.6%). It can be said that the webcam, microphone, and internet connection features with the highest frequency are the basic requirements for an exam to be safe. The reason why systems that require plug-in/browser/application installation are less preferred may be that not every user knows how to do them. Tüfekci et al. (2013) stated that online exam tools should be used without the need for additional computer skills.

On the other hand, although the majority of the online assessment systems examined stated that the users would need an internet connection, it was observed that no clear information was shared regarding the minimum download and upload speeds that the user should have for the system to work properly.

#### Integration methods used in online assessment systems

Online assessment systems can import and/or export data from/to an existing system. Table 12 shows integration methods used in prominent online assessment systems. Table 12 shows that the most supported integration methods in prominent online assessment systems are as follows: API (f = 13, 59.1%), plugin (f = 5, 22.7%), and LTI (f = 4, 18.2%). An API is an internet technology that enables different platforms to work in integration with each other and facilitates services. Learning Tools Interoperability (LTI) is an instructional technology standard supported by many LMS (ImsGlobal, 2021). LMS integration of online assessment systems is important for institutions (Sietses, 2016). At this point, it has been understood that more than half of the online assessment systems examined have integration with other systems, but in a few of them, students and exam information are defined by users. In future studies, new methods can be researched that will enable secure information sharing between online assessment systems and systems where student information is kept, which will be easy to integrate and have fast study performance.

**Table 12:** Integration methods used in online assessment systems

Integration methods	f	%
Application programming interface (API)	13	59.1
Plugin	5	22.7
Learning tools interoperability (LTI)	4	18.2
Online exam system engineers	1	4.5
Plug&Play	1	4.5
Instance url and secret key/authorization token	1	4.5
Unspecified	1	4.5

## Conclusions

This research aims to determine the trends in (1) supported platforms, (2) security features, and (3) common features of the online assessment systems.

The first research question has revealed that some of the existing online assessment systems are not mobile-friendly. However, the ability to attend assessments online via mobile devices can ensure flexibility in education. In addition, considering the socio-economic status of the students and mobile phones are more affordable than computers: it can be said that the rate of owning a mobile phone can be higher than the rate of having a computer. On the other hand, faculty can benefit from LMS integration while transferring data such as student identification and grade transfer. Therefore, developers should take into account that future online assessment systems would (i) provide mobile device support and (ii) enable e-Learning platform integration, and decision-makers would prefer the systems that have (iii) mobile device support and (iv) common web browser support that students frequently used.

The second research question helps to better understand the security features of the online assessment systems. The security features of these systems towards detecting and preventing cheating have been synthesized in this research, and it is understood that the majority of these systems authenticate users/students through government-issued IDs; disable copy/paste functions; use semi-automatic monitoring methods; and analyze the video, image, voice, and screen records. The findings synthesized in the previous section can guide decision-makers, researchers and system developers working on security in online assessment systems, and can help them to better understand what features should/will online assessment systems include in order to ensure security.

The third research question focuses on common features of the prominent online assessment systems, and synthesis of the common features has revealed the following characteristics: question types they support are multiple-choice, essay, and true/false questions; modules they include are frequently asked questions module, help documents module, and technical support module; requirements are webcam, microphone, and Internet connection; and data sharing method they support is API. Hereby, these features can be expressed as the basic features of an online assessment system and can be taken into account by future studies.

This research examines online assessment systems as a tool, and findings of this research (characteristics of online assessment systems) can guide practitioners, decision-makers, researchers, and system developers in the process of selecting and/or developing an online assessment system in the future. However, since students are the users of online assessment systems, it is important to understand students' views, how online assessment systems reflect on students and support their learning. Therefore, future studies can address the effects of online assessment systems on various academic factors.

### Limitations

The findings of this research are limited to the features of online assessment systems mentioned in the studies indexed by Google Scholar in 2020. The studies on online assessment systems were identified by using search queries in January 2021.

Various contradictory pieces of information were encountered on the web pages of some online assessment systems. Therefore, researchers sent the data collection form/tool to the online assessment system companies via email. However, if a company did not respond, then researchers collected data from its web page (including videos and help documents) and social media accounts. When contradictory or confusing statements were found in the data sources, the researchers filled in the data collection form by adding their comments.

## Data Availability

All data analyzed during this study are included in this published article (Table 1).

## Abbreviations

ID: Identity
LMS: Learning management system
OS: Operating system
PC: Personal computer
PRISMA: Preferred Reporting Items for Systematic Reviews and Meta-Analyses
